# Informed, advance refusals of treatment by people with severe mental illness in a randomised controlled trial of joint crisis plans: demand, content and correlates

**DOI:** 10.1186/s12888-017-1542-5

**Published:** 2017-11-24

**Authors:** Claire Henderson, Simone Farrelly, Clare Flach, Rohan Borschmann, Max Birchwood, Graham Thornicroft, Waquas Waheed, George Szmukler

**Affiliations:** 1 Health Service and Population Research Department, David Goldberg Centre, Institute of Psychiatry, Psychology & Neuroscience, King’s College London, De Crespigny Park, London, SE5 8AF UK; 2 Medical Statistics Unit, Health and Social Care Research, King’s College London, London, UK; 3 School of Psychology, University of Birmingham, Birmingham, UK; 4 Division of Psychiatry, School of Medicine, University of Manchester, Manchester, UK; 5 Centre for Adolescent Health, Murdoch Children’s Research Institute, Royal Children’s Hospital, Melbourne, Australia; 6 Centre for Mental Health, Melbourne School of Population and Global Health, University of Melbourne, Melbourne, Australia; 7 Department of Psychiatry, University of Melbourne, Melbourne, Australia

## Abstract

**Background:**

In the UK, crisis planning for mental health care should acknowledge the right to make an informed advance treatment refusal under the Mental Capacity Act 2005. Our aims were to estimate the demand for such treatment refusals within a sample of service users who had had a recent hospital admission for psychosis or bipolar disorder, and to examine the relationship between refusals, and service user characteristics.

**Methods:**

To identify refusals we conducted content analysis of Joint Crisis Plans, which are plans formulated by service users and their clinical team with involvement from an external facilitator, and routine care plans in sub-samples from a multi-centre randomised controlled trial of Joint Crisis Plans (plus routine mental health care) versus routine care alone (CRIMSON) in England. Factors hypothesised to be associated with refusals were identified using the trial data collected through baseline interviews of service users and clinicians and collection of routine clinical data.

**Results:**

Ninety-nine of 221 (45%) of the Joint Crisis Plans contained a treatment refusal compared to 10 of 424 (2.4%) baseline routine care plans. No Joint Crisis Plans recorded disagreement with refusals on the part of clinicians. Among those with completed Joint Crisis Plans, adjusted analyses indicated a significant association between treatment refusals and perceived coercion at baseline (odds ratio = 1.21, 95% CI 1.02–1.43), but not with baseline working alliance or a past history of involuntary admission.

**Conclusions:**

We demonstrated significant demand for written treatment refusals in line with the Mental Capacity Act 2005, which had not previously been elicited by the process of treatment planning. Future treatment/crisis plans should incorporate the opportunity for service users to record a treatment refusal during the drafting of such plans.

**Trial registration:**

ISRCTN11501328 Registered 13th March 2008.

## Background

Psychiatric crises, such as a relapse of psychosis, may be associated with reduced decision-making capability [1]. In the United Kingdom (UK), health service users have the right under the Mental Capacity Act (MCA) 2005 to make an informed advance treatment refusal [2] when capacitous, regarding times when their decision-making capability is impaired. Advance refusals are legally binding, but may be overridden if the patient is subject to an involuntary treatment order under the Mental Health Act 1983, amended 2007 (MHA). People subject to the MHA are given reasons for their detention and their rights are explained at the point of detention. In the case of advance refusals, an appropriate point at which to inform service users of the legal right to make such a refusal and help them to exercise it is during the process of crisis planning for mental health care.

In the national Health Service (NHS) mental health services in England, a written crisis plan is routinely present in the care plan, and is written and updated through the Care Programme Approach (CPA) [3]. This provides the current benchmark for written crisis plans. An audit of CPA forms showed very few documented refusals, at just 2.4% of a sample of 424 crisis plans [4]. In contrast, studies of both facilitated psychiatric advance directives (f-PADs) in the US [5, 6] and Joint Crisis Plans (JCPs) in the UK [7, 8] show much higher rates of refusals, at around three quarters of participants completing a f-PAD [5] or JCP [8]. These are structured research interventions in which an independent mental health professional facilitates a discussion about preferences for future care - including treatment refusals - with the service user and, in the case of the JCP, with professionals involved in his or her care [9]. The discrepancy between these interventions and routine care plans suggests that if service users are given better information about their rights, more would make advance refusals. However, the previous studies in the UK occurred before the MCA 2005 was introduced, so it is not clear whether the situation concerning refusals has changed. One trial conducted since the introduction of the MCA 2005 of JCPs for outpatients with borderline personality disorder [10] showed a very high (90.2%) rate of refusals, but no comparison with the rate of refusals in the conventional treatment plan was made.

We therefore made use of data from a recent multisite trial of JCPs [11–13] that incorporated and explained the relevant provision in the MCA. Our first aim was to ascertain whether - and to what extent - the JCP process elicited more written advance treatment refusals than the CPA process during the process of a pragmatic randomised controlled trial. Our second aim was to explore the nature of such refusals and the relationships between making a refusal with service user characteristics.

To address the first aim we tested the hypothesis that the rate of refusals would be higher in JCPs than in CPA treatment plans. To address the second aim we tested the following hypotheses: (1) the majority of refusals will be accepted by clinicians without concerns that they will reduce the standard of care provided/received [6]; (2) refusals are more likely to be made by participants who either a) have prior experience of coercion i.e. transport or detention under the Mental Health Act; b) report a high level of perceived coercion at baseline; or c) report poor working alliance with professionals at baseline. All of these factors may indicate a lack of acceptance of at least some aspects of treatment and hence a wish to avoid these treatment components in future.

## Methods

### Setting and sample

The sample for this study is a sub-sample from the CRIMSON trial [11–13]. The CRIMSON trial was a multi-site randomised controlled trial of JCPs for individuals with psychotic disorders in four mental health Trusts (NHS service provider organisations) in the UK (total *n* = 569). The eligibility criteria for the CRIMSON trial were: diagnosis of a psychotic disorder; admission to a psychiatric ward in the last two years and current contact with a community mental health team. Service users who were in hospital or under a section of the Mental Health Act at the time of recruitment were deemed ineligible so as to avoid any potential perceived coercion to participate. No other exclusions were made, thus maximising generalisability. This study uses data from three overlapping study samples: (1) intervention group participants who completed the JCP process (*n* = 221) and whose JCP could therefore be assessed for refusals; and (2) the 424 patients whose CPA care plans were available for assessment for treatment refusals at baseline; and (3) those in the control group whose CPA care plans were available at baseline and follow up (*n* = 221).

### The intervention

The JCP [7, 14] was developed in the later 1990’s. The starting point was a review of existing models containing information about the service user and requests for treatment during a relapse or crisis [14]. Sections were then added to encourage reflection about past crises that would help inform the thinking and planning for future crises. Headings were included where circumstances or triggers for past relapses could be recorded, as well as details of early signs of relapse and space to record both the good and bad aspects of care received during previous crises. These headings precede the advance planning section so that during the meeting to finalise the plan these aspects are discussed. Eliciting this information promotes a process of shared decision making at the planning meeting, as the treating clinicians are present and can also share their observations of early warning signs, triggers, and what has been helpful/not helpful in the past [15]. The completed JCP contains the service user’s statements about past treatment and preferences for care in the event of a future relapse or crisis. It was produced after two meetings. At the first meeting, the JCP facilitator - a member of the research team independent of the clinical team - explained the procedure to the service user and the care coordinator. To finalise each plan, the service user was encouraged to bring a carer, friend, or advocate to a second meeting. The aim of this meeting was to discuss the views of the service user, his or her informal supports(s) and treating professionals regarding what to do in a future crisis, and to negotiate agreed solutions. The JCP facilitator’s role was to ensure that everyone’s perspective was heard but the final choice of what was included in the plan (including the exact terminology used) lay with the service user. The clinical team was present to discuss options and the implications of the service user’s choice. When the option of stating which treatment(s) they did not want to receive in future was discussed, the facilitator explained the MCA 2005 provision for making a treatment refusal. All JCPs were written in the first person to reflect the service user’s own description of their mental health issue and wishes for treatment. At the end of this meeting the JCP facilitator asked clinicians if they were happy to agree to the overall plan, in order to clarify to the service user any content that they thought could not be followed and have this acknowledged on the JCP.

After finalising the JCP, service users were given a copy and an electronic version of the plan was stored on the Trust’s electronic patient record system. Once completed, the content of the JCP could be updated by service user and the regular clinical team and used in routine care planning. The intervention group received the JCP intervention in addition to treatment as usual (described below). As the unit of randomisation was the service user, some individual care coordinators and individual doctors may have developed more than one JCP. Likewise, some individual care coordinators and doctors may have had service users in both the control and intervention groups.

### Treatment as usual control

The control condition was Treatment As Usual as stipulated under the CPA. Treatment As Usual under the CPA includes requirements that all service users are assessed, receive a written care plan that includes a crisis and contingency plan, and that this is reviewed regularly. In most situations, service users have a nominated Care Coordinator, whose role is to be the central point for communication regarding the service user and to ensure the service user’s identified health and social care needs are met. Treatment As Usual thus in theory allows the making of refusals and their documentation on the CPA form by the Care Coordinator, although the form may not specifically prompt them to do so. Thus, the control condition not only lacks the process of facilitation but also depends on existing documentation required by the service.

### Assessments

Assessment for treatment refusals and whether clinicians would not be able to follow them: Refusals were assessed in both the JCPs and CPA care plans. A content analysis of the JCPs was conducted by SF. Refusals were coded as present or not present, and content of refusals was thematically coded [16]. For refusals on the CPA care plan, the entire CPA care plan was printed and anonymised by research assistants. SF assessed the content of each plan at baseline and follow-up for the presence of treatment refusals and for the presence of statements by clinicians about any aspect of the JCP that they thought could not be followed/adhered to.

Demographics: Research assistants collected demographic data from service users and Care Coordinators including age, sex, ethnicity, education/qualification and number of years in contact with mental health services. For Care Coordinators, length of psychiatric practice and length of relationship with the service user were also collected.

Hospitalisations: Data regarding psychiatric admissions in the two years prior to baseline and over the follow-up period including duration and admission status (formal/informal) were collected by research assistants from routine Trust data sets and corroborated by service users and clinicians at interview.

Self-harm and harm to others: At baseline interview participants were asked about instances and severity of self-harm, suicide attempts and harm to others in the two years prior to baseline and, at follow-up, they were asked about the period since baseline assessment (usually 18 months).

*Objective Coercion:* use of the Mental Health Act to transport the service user or detain them in a psychiatric unit.

*Perceived Coercion:* was measured by the perceived coercion subscale of the service user self-report measure the MacArthur Perceived Coercion Scale [17], adapted for use in outpatient treatment. Higher scores indicate more perceived coercion.

*Therapeutic Relationships:* The Working Alliance Inventory short form (WAI-S) [18, 19] modified for use in psychiatric samples [20] was rated by service users and clinicians (WAIC and WAIT respectively) at baseline and follow-up.

*Engagement:* was measured by the Service Engagement Scale (SES) [21]. This is a 14-item scale producing four subscales measuring ‘availability’, ‘collaboration’, ‘help seeking’ and ‘treatment adherence’ and a total score. Higher scores on this measure indicate poorer engagement. This measure was rated by the Care Coordinator.

### Data analysis

To assess the proportion of refusals not identified through the CPA process we compared the proportion of refusals in the 221 completed JCPs with that in CPA forms available: (1) at baseline for the entire group (*n* = 424); and (2) at follow-up for the control group only (*n* = 203), as the presence or absence of a refusal in a JCP could influence whether it was present in the participants’ CPA form at follow-up. The analyses aimed at identifying factors associated with refusals were restricted to the JCP group as it was only in this group that there was a sufficient number.

Associations between covariates and missing data regarding treatment refusal were investigated using chi-squared and t-tests as appropriate. Covariates found to be associated with missing data were accounted for in all analyses. Chi-squared analyses and t-tests were performed to ascertain any relationships between making a refusal and baseline service user clinical or demographic characteristics based on bivariate analysis. The hypothesised factors associated with a refusal were: previous compulsory admissions, perceived coercion and service user-rated working alliance at baseline. These were regressed on an indicator of refusal of treatment using logistic regression. The logistic regression model was adjusted for covariates associated with missing information or treatment refusal.

## Results

### Rates of refusals in the CPA forms versus JCPs

Of the total sample for the CRIMSON trial (*n* = 569, 284 control, 285 intervention), 424 (74%) CPA forms were available (221 controls and 203 intervention). We were unable to obtain records from one Trust (*n* = 48). The remainder were missing due to: not being able to locate a care plan at baseline or follow-up (*n* = 52); participants being discharged at follow-up (*n* = 22) or downgraded to care support and thus no care plan (*n* = 8); deaths (*n* = 5); and refusing access to records (*n* = 10). Ten of these 424 CPA forms (2.4%) included refusals at baseline. For the control group, six of the 221 (2.7%, 95% CI 1.0%–5.8%) included a refusal at follow-up on their CPA forms. In the intervention group 221/285 (77%) completed a JCP. Of these, 99 (45%, 95% CI 38%–52%) made a refusal as part of the JCP.

### Agreements with JCPs by clinicians

There were no cases where clinicians explicitly disagreed with the final version of the JCP.

### Types of refusal

Based on the JCP sample, the most common refusal was regarding aspects of medication: 45/99 (45%) made a refusal of a specific medication (with 53% of this subsample providing a reason for this refusal); 20/99 (20%) refused injections. Others made refusals regarding high doses of medication, increases or changes to dose. Only one participant stated that they would prefer not to take any medication. Seventeen refusals regarding specific medications were made: one anti-cholinergic, three different mood stabilisers, two anti-depressants, one anti-anxiolytic and ten anti-psychotics.

The most frequently refused anti-psychotics were: haloperidol (*n* = 9), olanzapine (*n* = 8), risperidone (*n* = 5), zuclopenthixol (*n* = 5), quetiapine (*n* = 4) and chlorpromazine (*n* = 4). The next most common category of refusal was electro-convulsive therapy (ECT; 19/99 (19%). Eighteen participants (18%) made refusals relating to admission to hospital. Of these, eight (44%) made refusals about specific aspects of admission, such as which ward they were admitted to. The remaining ten (56%) refused hospital admission, though seven added a caveat, for example, “[I refuse] being put in hospital. I know that this is sometimes necessary however I would like this to be the last resort”. While these caveats reflect acknowledgements of the views of clinicians, there was no indication on the other three as to whether clinicians disagreed with these refusals.

### Predictors of refusal

Sixty-four (22%) of participants in the JCP arm did not complete a JCP. These participants were similar in most respects to those who did, but tended to have had more psychiatric admissions in the two years prior to the trial, have a higher engagement with care (SES) score and higher clinician-rated working alliance (WAIT). We therefore adjusted further analyses for the number of admissions, SES and WAIT score to account for bias due to missing data.

Table 1 describes the sample with JCP refusal information by refusal status. Treatment refusal was individually associated with the participant’s location, ethnicity, years in contact with mental health services and the perceived coercion subscale of the McArthur coercion scale before adjustments. There was no bivariate association between having had an involuntary admission in the two years prior to baseline assessment or service user-rated working alliance and treatment refusal.

**Table 1:** Description of the JCP sample at baseline by refusal status

Variable	Category Value	Total *N* = 221	Treatment Refusal *N* = 99	*p*-value
Site	London	77	18 (23%)	
	Birmingham	75	42 (56%)	
	Manchester/Lancashire	69	39 (57%)	< 0.001
Sex	Male	113	48 (42%)	
	Female	108	51 (52%)	0.398
Age	mean (sd)	40.4 (11.5)	41.2 (11.6)	0.375
Ethnicity (grouped)	White-all	141	76 (54%)	
	Black/Black British - all	51	13 (25%)	
	Other	29	10 (35%)	0.001
Education	None	65	31 (48%)	
	School	103	41 (40%)	
	Vocational	24	14 (58%)	
	Higher	27	12 (44%)	0.386
Diagnosis OPCRIT grouped^a^	Schizophrenia spectrum disorders	162	70 (43%)	
	Affective disorders	59	29 (49%)	0.432
Clinical measures over last two years				
Number of admissions	mean (sd)	1.47 (0.92)	1.37 (0.71)	0.181
A formal Admission	No	91	40 (44%)	
	Yes	130	59 (45%)	0.768
Self-harm	No	175	81 (46%)	
	Yes	46	18 (39%)	0.385
Suicide attempt	No	162	73 (45%)	
	Yes	59	26 (44%)	0.895
Harm to others	No	196	88 (45%)	
	Yes	23	10 (43%)	0.897
Years in contact with MHS	mean (sd)	14.6 (9.4)	16.0 (9.9)	0.041
Perceived Coercion (MacArthur)		2.25 (1.6)	2.54 (1.5)	0.016
Engagement with care (SES)		9.21 (6.9)	8.74 (6.8)	0.397
Therapeutic relationship:	Self-rated (WAIC)	15.8 (6.3)	16.2 (6.3)	0.404
	Therapist-rated (WAIT)	16.7 (5.0)	17.0 (5.2)	0.483

^a^ OPCRIT [31]

Logistic regression of the refusal status on the three hypothesised baseline predictors (involuntary admission, perceived coercion and therapeutic alliance) adjusting for site, ethnicity, contact with services and covariates associated with missingness (SES, number of admissions and carer-rated working alliance) are provided in Table 2. The results indicate that there is no association between working alliance or involuntary admission and advance treatment refusals. The association between perceived coercion and advance refusals remains significant when other factors are accounted for (odds ratio = 1.34, 95% CI 1.07–1.69, *p* = 0.013). Table 2 also shows that participant location remains associated with advance refusals after adjustment for other factors.

**Table 2:** Hypothesised baseline predictors of treatment refusal within the JCP treatment arm, adjusted for covariates listed, *n* = 168

Covariate	Odds Ratio	95% Confidence Interval		p-value
Formal admission	1.42	0.65	3.11	0.383
Perceived Coercion	1.34	1.07	1.69	0.013
Therapeutic relationship service user-rated (WAIC)	1.00	0.94	1.06	0.877
Number of admissions in past 2 years	0.84	0.55	1.28	0.409
Engagement with care (SES)	0.95	0.89	1.02	0.154
Therapeutic relationship clinician-rated (WAIT)	1.02	0.93	1.12	0.687
Site: Birmingham vs. London	6.19	2.37	16.19	< 0.001
Manchester/Lancashire vs. London	5.94	2.19	16.12	< 0.001
Ethnicity: Black/Black British vs. White	0.57	0.20	1.67	0.307
Other vs. White	0.54	0.19	1.56	0.255
Years in contact with MHS^a^	1.04	1.00	1.08	0.060

^a^mental health services

## Discussion

### Summary of findings

Comparison of the rates of advance refusals made in JCPs with those made in CPA forms among trial participants shows that the potential demand for refusals is barely addressed by the current CPA process used in routine care. Our results also suggest that most refusals made using the JCP were not out of keeping with what the clinicians viewed as safe clinical practice. In no cases did clinicians attending the JCP meetings disagree with the refusals made, although it should be noted that this does not mean all refusals were then followed in practice. It is also possible that clinicians did not voice their concerns during the meetings (despite being asked for their views by the facilitator), as suggested by qualitative interviews of some participating clinicians [22]. They may instead have wanted to avoid an argument with the service user and/or have judged the process to be non-binding. Thus, knowing that refusals made under the Mental Capacity Act 2005 can be overridden using the Mental Health Act may reduce the attention paid by clinicians to refusals made by serviced users, despite the possibility of honouring a refusal (such as that of a particular medication) while someone is detained under the Mental Health Act. Among those who received a JCP, higher perceived coercion at baseline was associated with subsequent treatment refusal, as hypothesised. However, no association was found with having a previous involuntary admission or working alliance and subsequent treatment refusal.

The association with duration of contact with mental health services, along with the lack of association with involuntary admission or working alliance, suggests that many refusals are made based on experiences of treatments regardless of whether these were experienced while detained under the Mental Health Act. Participant location at the sites outside London was also associated with treatment refusal. This may indicate differences in implementation of the JCP development process among the sites on the part of the facilitators, despite the provision of supervision of all facilitators by the same member of the research team. A second possibility is the existence of site level differences in participants’ past experiences of treatment, which were not captured in the assessment and exclude use of the Mental Health Act per se, but which give rise to higher rates of treatment refusal.

### Limitations of the study

While the JCP intervention allowed us to assess demand for treatment refusals and their relationship with clinical and demographic factors, the sample was subject to the eligibility criteria for the CRIMSON trial. This took place in three urban and one rural area within the three sites and included only those with an admission in the last two years; the sample is therefore not fully representative of adults with psychoses using secondary mental health services in England. Further, we are unable to report the extent to which refusals were honoured due to the CRIMSON trial resource limitations.

## Conclusions

Our findings are consistent with previous research showing there is strong demand for psychiatric advance statements [23–25] and that the content of most advance statements can be followed without compromising care [6, 25, 26]. However, most of those who want to make a statement need support to complete it [5, 27], and despite the existence of UK legislation for advance refusals, services offer no systematic or efficient way of providing this support. We have thus highlighted an aspect of the Mental Capacity Act 2005 which has been under-implemented in mental health services. The creation of, access to, and honouring of individualised crisis plans which include refusals are important in their own right but are missing from current care pathways [4].

The research evidence is clear that interventions such as Joint Crisis Plans and facilitated psychiatric advance directives are an effective way to inform service users of their legal rights to make advance treatment refusals; they probably also improve working alliance with clinicians [13, 24]. Evidence that these interventions can lead to ‘harder’ outcomes such as reduced use of involuntary treatment [28] and for their cost-effectiveness [12, 29] is more mixed, and may reflect problems in implementation.^11^ However, a recent systematic review identified advance statements as the only intervention associated with reduction in the use of compulsory treatment [28]. Further work is needed to identify ways to overcome barriers to the creation of advance statements, together with study of the outcomes associated with not just their creation but of their application in routine practice.

Recently arguments have been presented that involuntary detention and treatment under mental health legislation be like that for general health care, that is, based on impaired decision-making capacity and best interests [30]. In such a case, a treatment refusal could not be ‘trumped’ by mental health legislation. If the law were to be reformed, the patient’s decision-making capacity would need to be assured, especially when a treatment refusal with serious health consequences was desired. The place of a state’s duty to protect life, as under the European Convention on Human Rights, might need to be considered. The impact of legal reform on the implementation of support for service users to make advance statements, which may include refusals, is not clear.

## Abbreviations

CPA: Care Programme Approach
f-PAD: facilitated Psychiatric Advance Directive
JCP: Joint Crisis Plan
MCA: Mental Capacity Act 2005
MHA: Mental Health Act 1983, amended 2007
SES: Service Engagement Scale
WAIC: Working Alliance Inventory short form Client version
WAI-S: Working Alliance Inventory short form
WAIT: Working Alliance Inventory short form Therapist version
